# Proxy Molecular Diagnosis from Whole-Exome Sequencing Reveals Papillon-Lefevre Syndrome Caused by a Missense Mutation in *CTSC*

**DOI:** 10.1371/journal.pone.0121351

**Published:** 2015-03-23

**Authors:** A. Mesut Erzurumluoglu, Muslim M. Alsaadi, Santiago Rodriguez, Tahani S. Alotaibi, Philip A. I. Guthrie, Sian Lewis, Aasiya Ginwalla, Tom R. Gaunt, Khalid K. Alharbi, Fahad M. Alsaif, Basma M. Alsaadi, Ian N. M. Day

**Affiliations:** 1 Bristol Genetic Epidemiology Laboratories (BGEL), University of Bristol, Oakfield House, Oakfield Grove, Bristol, BS8 2BN, United Kingdom; 2 College of Medicine, Pediatric Department, King Saud University, P.O. Box 50807, Riyadh, 11533, Kingdom of Saudi Arabia; 3 MRC Integrative Epidemiology Unit (IEU), School of Social and Community Medicine, University of Bristol, Oakfield House, Oakfield Grove, Bristol, BS8 2BN, United Kingdom; 4 Clinical Laboratory Sciences Department, College of Applied Medical Sciences, King Saud University, P.O. Box 10219, Riyadh, 11433, Kingdom of Saudi Arabia; 5 Department of Dermatology, King Saud University, P.O. Box 7805, Riyadh, 11472, Kingdom of Saudi Arabia; Institut Jacques Monod, FRANCE

## Abstract

Papillon-Lefevre syndrome (PLS) is an autosomal recessive disorder characterised by severe early onset periodontitis and palmoplantar hyperkeratosis. A previously reported missense mutation in the *CTSC* gene (NM_001814.4:c.899G>A:p.(G300D)) was identified in a homozygous state in two siblings diagnosed with PLS in a consanguineous family of Arabic ancestry. The variant was initially identified in a heterozygous state in a PLS unaffected sibling whose whole exome had been sequenced as part of a previous Primary ciliary dyskinesia study. Using this information, a proxy molecular diagnosis was made on the PLS affected siblings after consent was given to study this second disorder found to be segregating within the family. The prevalence of the mutation was then assayed in the local population using a representative sample of 256 unrelated individuals. The variant was absent in all subjects indicating that the variant is rare in Saudi Arabia. This family study illustrates how whole-exome sequencing can generate findings and inferences beyond its primary goal.

## Introduction

Papillon-Lefevre syndrome (PLS, MIM# 245000) is an autosomal recessive disorder characterised by severe early onset periodontitis and palmoplantar hyperkeratosis, which consequently results in the premature loss of the primary and secondary dentitions [1]. PLS is caused by mutations in the *CTSC* gene which displays remarkably high allelic heterogeneity with over 70 mutations reported hitherto [2]. *CTSC* encodes the cathepsin C protein, a lysosomal exo-cysteine proteinase belonging to the peptidase C1 family [2].

We had investigated the family previously for a Primary ciliary dyskinesia (PCD) study where the whole-exome of the two children with PCD was sequenced (though the PCD causal variant remains unknown). However, family history indicated that there were two other siblings diagnosed with PLS and the family requested a molecular diagnosis for this disease also. We reasoned that with a one-third chance of each PCD (PLS unaffected) sibling with whole-exome sequencing (WES) data being a non-carrier for the PLS causal mutation, we would have an 8/9 chance (1/3 x 1/3 = 1/9 = chance of both **not** being a carrier) of identifying the PLS causal mutation (likely in *CTSC*) in a heterozygous state in at least one of the two available WES data. This paper describes our findings. We also discuss the ethical and research implications of this study.

## Materials and Methods

### Ethics Statement: Approval and consents

Ethical approval was obtained from the King Saud University/King Khalid Hospital, Riyadh ethics committee (approval number: E-11–448). Family and individual consent was written, with the recognition that positive findings would be diagnostically reconfirmed in conjunction with clinical counselling and feedback. Consent was obtained following family/patient information session (initially through telephonic conversation, and then by recap in clinic). Written parental consent was obtained for minors. The information obtained from the collective family visit to clinic is also added to hospital clinical notes as is the record of any buccal samplings for DNA agreed and undertaken. For this family, parental consent was from the father: both parents and their children attended the clinic together.

As aforementioned, the participating family was previously analysed for a PCD study [3,4]; and this study was carried out *after* the family re-attended the clinic enquiring about the cause of PLS present in other siblings within the family.

A local population reference DNA sample (*n* = 256) was set up, comprising of male and female student volunteers of Saudi Arabian ancestry at the King Saud University (Riyadh, Kingdom of Saudi Arabia). Inclusion for the mutation screening process was voluntary. The informed written consent of these individuals for anonymised genetic studies was taken in keeping with King Saud University College of Applied Medical Sciences guidelines.

### Participants and Genetic Data Analysis

A male proband from a consanguineous family of Arabic descent with clinical features consistent with PLS including loss of primary teeth and nail dystrophy was analysed. Additionally, three siblings’ (one affected and two unaffected, including the PCD affected) and the parents’ blood samples were also collected for further analysis. DNA was extracted from peripheral blood samples using the QIAamp DNA Mini kit provided by QIAGEN (Catalogue No: 51304) using their protocol for “DNA Purification from Blood or Body Fluids”. The exome of the PCD affected sibling was captured using the Agilent SureSelect Human All Exon 50M exon capture kit (Agilent Technologies, Inc. Santa Clara, CA, 95051, USA) and WES data was obtained by subsequent sequencing using the Illumina Hiseq2000 platform (Illumina, Inc. San Diego, CA, 92122, USA). The Burrows-Wheeler Aligner (BWA) [5] software was used to align the reads to the latest human genome reference sequence (hg19), filtering out reads which have extensive low base quality (more than half of the bases which have a base quality of ≤ 5, including no calls) and/or with a mapping score of zero. Picard (http://picard.sourceforge.net) was used to mark duplicated reads and the alignment results were generated in BAM format. Single nucleotide polymorphisms (SNPs) were called using SOAPsnp [6] and small insertion/deletion events (indel) were detected by SAMtools and GATK, and exported in VCF format [7–9]. VCF annotations were obtained from the Ensembl Variant Effect Predictor (VEP) [10] and ANNOVAR [11]. Predictions for missense mutations were obtained from FATHMM [12], SIFT (via VEP) [13], Polyphen-2 (via VEP) [14] and Condel (via VEP plugin) [15]. The *CTSC* gene was screened for variants which are either rare (<0.1%) or absent in the 1000 Genomes project [16] and Exome Variant Server (EVS) [17].

### PCR amplification and Sanger sequencing

Region specific primers (i.e. where the NM_001814.4:c.899G>A:p.(G300D) variant is located) were designed and PCR (annealing temperature: 49C°) was used to amplify a 220bp long region containing the c.899G>A:p.(G300D) variant in the parents and the siblings (Forward primer: 5'- AAGCTAAGAACAACTTTCAGGG-3' and Reverse primer: 5'- TGGAGAATCAGTGCCTGTGTAG-3'). These amplicons were purified and subsequently sequenced using Sanger sequencing.

### Screening for the c.899G>A:p.(G300D) variant in the local population

DNA was extracted from 256 unrelated (and healthy) individuals living in Riyadh using methods described above. Four primers (Control forward primer: 5'-AACATGCAAAGAATAATGGAG-3', Common reverse primer: 5'-AGCTTCATCAGGGCTTCATTG-3', Mutant allele-specific primer: 5'-TTCATCTTCAGGCTGTGAACG-3' and Wild-type allele-specific primer: 5'-TTCATCTTCAGGCTGTGAACA-3') were designed (see S2 Table for the primers used in ARMS-PCR for genotyping the variant) and ARMS-PCR (annealing temperature: 47C°, see Gaunt *et al*., 2001 for description of method [18]) was used to detect the presence of the c.899G>A:p.(G300D) variant in 256 unrelated individuals selected from the local population in Riyadh. Resulting PCR amplicons were then viewed using 96-well microplate array diagonal gel electrophoresis (MADGE) [19]. The same procedure was also repeated on the family members to ensure validity of the method. Nucleotide numbering system uses +1 as the A of the ATG translation initiation codon in the reference sequence, with the initiation codon (Met) as codon 1.

## Results

### Whole-exome sequencing of PCD affected sibling

The total length of all captured regions was 118,361,446 base pairs (50,599,905 bases on target and 67,761,541 bases near target, the latter being flanking regions within 200bp of exons). Coverage of target (i.e. exons) and flanking regions (e.g. introns, splice sites) was 98.2% and 92.5% respectively. The average sequencing depth on target was 60.75 and the fraction of target covered with at least 20, 10 and 4 reads was 79.4%, 88.6% and 94.6% respectively. There were a total of 51,084,667 (high quality) reads with a mapping rate of 99.39%.

### Identifying the PLS causal gene

**Fig 1:**
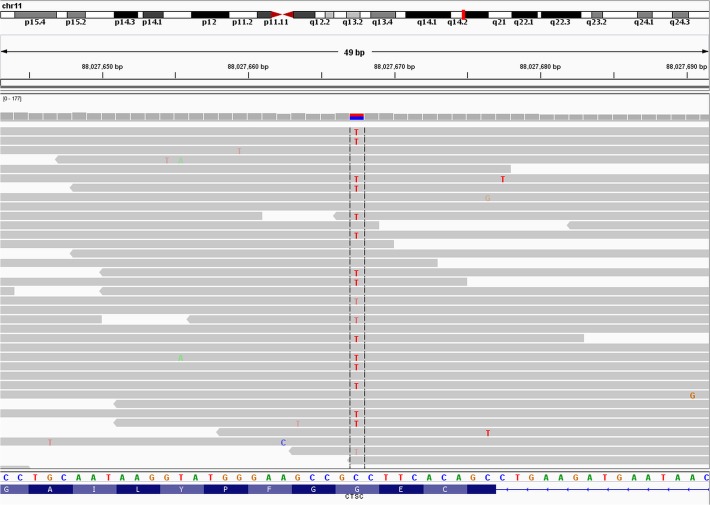
Reads (from PCD affected sibling) mapped to the human genome hg19 at the p.(G300D) mutation. The read depth is 46 (not all shown due to space restrictions) with twenty six of the reads having a G at the loci and twenty having an A. The image was created using IGV [20]. NB: The *CTSC* gene is oriented the reverse strand, therefore the codon change p.G300D (GGC>GAC) is exhibiting as C>T.

Whole-exome sequencing of the PCD affected sibling had previously been carried out (although no mutation causal of PCD has yet been identified) and her *CTSC* gene was analysed in follow up to the PLS presentation of two of her siblings [1]. 8 single nucleotide variations (intronic variants: rs217116, rs217060, rs580743, rs217075, rs217076, rs217077; missense mutations: rs217086 and c.899G>A:p.(G300D)) and a single nucleotide insertion (rs11426721) were identified in the *CTSC* gene. All except c.899G>A:p.(G300D) had a minor allele frequency of over 7% in the 1000 Genomes Project and EVS (see Fig. 1 for alignment of reads) which are too common to be causal of a rare Mendelian disease such as PLS. FATHMM (damaging, -3.06), SIFT (deleterious, 0.01), Polyphen (probably damaging, 0.998) and ConDel-2 (deleterious, 0.880) all predicted the c.899G>A:p.(G300D) variant to be functionally disruptive.

**Fig 2:**
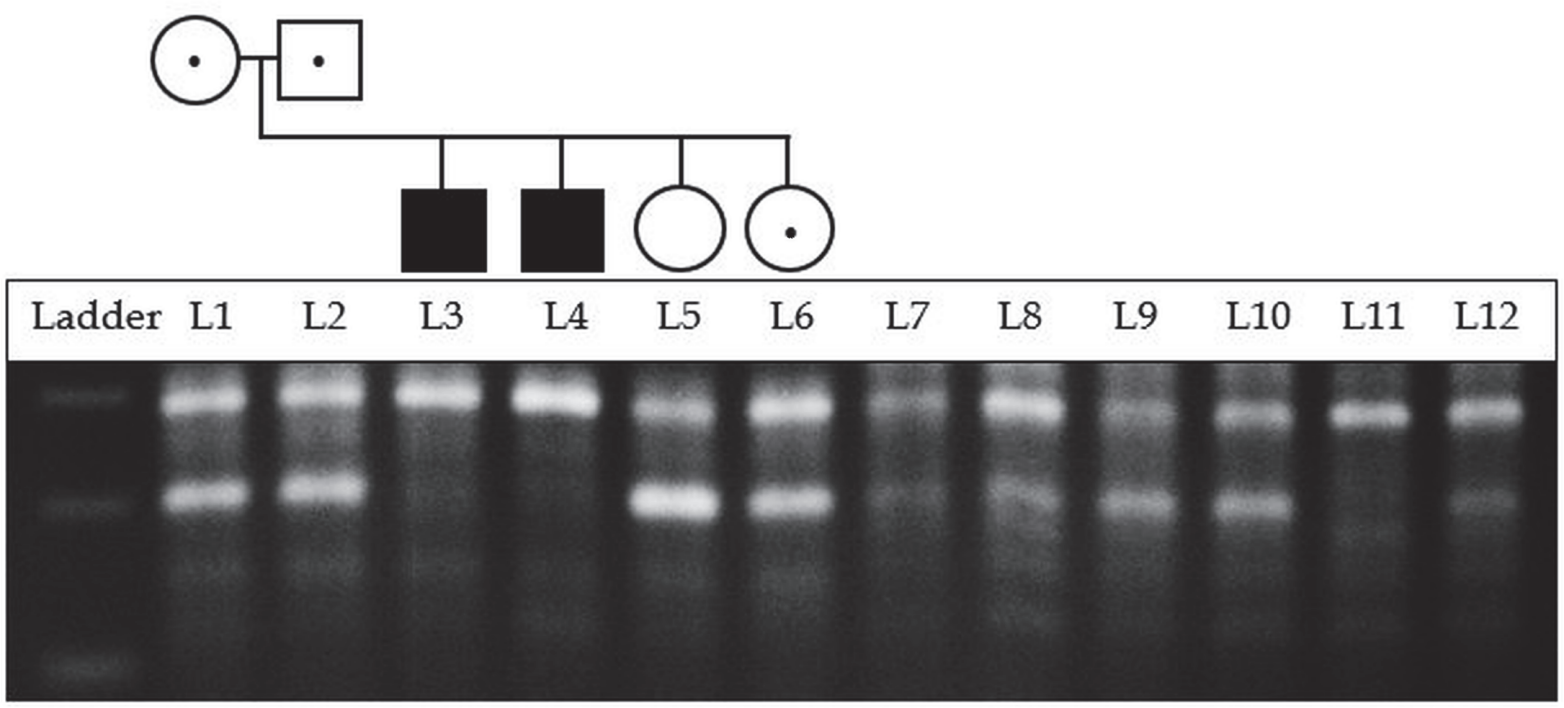
Validation of NM_001814.4:c.899G>A:p.(G300D) in all family members using ARMS-PCR. For primers, see S2 Table. L1-L6: using AS primer for wild type. L7-L12: using AS primer for mutant. L1/7: Mother. L2/8: Father. L3/9: PLS Proband. L4/10: PLS Affected brother. L5/11: Unaffected sibling—homozygous for *CTSC* wild type allele. L6/12: PCD affected sibling who is a carrier for PLS. Ladder’s three bands are 100bp (bottom), 200bp and 300bp (top).

The mutation also resides in a highly conserved region represented by a (36-way eutherian mammals) high GERP score of 1285.8 (also see S1 Table for local sequence alignment with other species) [21]. Searching the public mutation databases and the literature about the variant showed that it was previously identified in a homozygous state by Zhang *et al* in a single Saudi Arabian proband [22] and the variant was present in HGMD (Public version, ID: CM002939) and PhenCode (ID: CTSCbase_D0022:g.44271G>A) [23]. This provided strong evidence that this was the likely causal variant in the two PLS siblings. Thus the region containing the variant was amplified and sequenced using Sanger sequencing in both PLS affected siblings and the parents to confirm their status. In accordance with autosomal recessive mode of inheritance of PLS, the parents were heterozygous and the affected subjects were homozygous (see S1 Fig. for confirmation of variant status in other family members using Sanger sequencing). The other PLS unaffected sibling was homozygous for the wild type allele. ARMS-PCR was also used in all family members to establish mutation status (Fig. 2).

### Frequency of c.899G>A:p.(G300D) in Saudi Arabia

Allele-specific (AS) PCR amplicons (using primers in S2 Table) from the 256 participants were separated using 96-well MADGE (procedure was repeated three times). None showed the 207bp band characteristic of the mutant allele, whereas the band characteristic of the wild-type allele was present in all participants when wild type AS primer was used (S2A–F Fig. for the six 96-well MADGE images which show that none of the 256 individuals have the causal allele). The results for all 6 family members are shown in Fig. 2.

## Discussion/Conclusion

The *CTSC* gene displays high allelic heterogeneity and over 70 variants have been shown to cause PLS [2]. The c.899G>A:p.(G300D) variant is one of those, previously being reported in a single proband by Zhang *et al* [22]. Our findings follow up their paper as we have replicated their results, confirming the highly penetrant nature of the variant, and found that the prevalence of the variant in Riyadh, Saudi Arabia is rare (0 out of 512 chromosomes analysed). We also present a straightforward and cost-effective assay to test for this mutation.

The c.899G>A:p.(G300D) variant was identified in a previously whole-exome sequenced and PLS unaffected sibling of the proband which shows how additional inferences can be made from WES (i.e. proxy molecular diagnoses). Although WES targets only the coding regions of the genome (i.e. exome), it is thought to capture ∼85% of Mendelian disease-causal mutations [24]. Thus, where WES (or whole genome sequencing) data is available and consent is given, it can be a pragmatic choice to screen for known mutations using databases such as HGMD (Public and Paid versions available), PhenCode (Public) and ClinVar (Public).

However, there are ethical issues surrounding incidental findings [24,25]. WES data can be a source for these findings as it provides a pool of all detected variants in *all* genes. Therefore informed consent and abiding by the consent obtained is crucial (see [24] for a discussion on the matter). Our finding however, was not incidental and the study was carried out *only* after the family had attended the clinic with a second disorder (i.e. PLS) and gave consent for the subsequent analysis. We did not screen the family’s previously available WES data other than for previously known/suspected PCD causal variants (in accordance with previous consent) before we were given further consent to search for the PLS causal variant. The *CTSC* gene was then screened using the available WES data and a missense variant which was previously reported as PLS causal was identified in a heterozygous state in one of the PCD affected siblings [22]. This then enabled us to make a proxy molecular diagnosis and confirm the variant’s homozygosity status in the PLS affected siblings.

Our study highlights the wider and longer-term value of sequence data in the context of family history and additional clinical data. If it is stored and easy to query, it provides considerable potential for future diagnostics within families at minimal additional cost. In this example, once the PLS was diagnosed in the proband it took only a few minutes before the causal variant was identified in the PCD affected sibling whose WES data was available—saving considerable time, effort and cost.

## Supporting Information

S1 Fig Confirmation of variant status in other family members using Sanger sequencing. (DOCX)

S2 Fig Six 96-well MADGE images which show that none of the 256 individuals have the causal allele. (DOCX)

S1 Table Local sequence alignment containing the mutated residue from multiple alignment of the *CTSC* gene in different species. (DOCX)

S2 Table Primers used in ARMS-PCR for genotyping the NM_001814.4:c.899G>A:p.(G300D) variant. (DOCX)

## References

[1] Hart TC, Hart PS, Bowden DW, Michalec MD, Callison SA, Walker SJ, et al Mutations of the cathepsin C gene are responsible for Papillon-Lefevre syndrome. J Med Genet. 1999;36: 881–887. 10593994 PMC1734286

[2] Nagy N, Valyi P, Csoma Z, Sulak A, Tripolszki K, Farkas K, et al CTSC and Papillon-Lefevre syndrome: detection of recurrent mutations in Hungarian patients, a review of published variants and database update. Mol Genet Genomic Med. 2014;2: 217–228. 10.1002/mgg3.61 24936511 PMC4049362

[3] Alsaadi MM, Gaunt TR, Boustred CR, Guthrie PA, Liu X, Lenzi L, et al From a single whole exome read to notions of clinical screening: primary ciliary dyskinesia and RSPH9 p.Lys268del in the Arabian Peninsula. Ann Hum Genet. 2012;76: 211–220. 10.1111/j.1469-1809.2012.00704.x 22384920 PMC3575730

[4] Alsaadi MM, Erzurumluoglu AM, Rodriguez S, Guthrie PA, Gaunt TR, Omar HZ, et al Nonsense mutation in coiled-coil domain containing 151 gene (CCDC151) causes primary ciliary dyskinesia. Hum Mutat. 2014;35: 1446–1448. 10.1002/humu.22698 25224326 PMC4489323

[5] Li H, Durbin R. Fast and accurate short read alignment with Burrows–Wheeler transform. Bioinformatics. 2009;25: 1754–1760. 10.1093/bioinformatics/btp324 19451168 PMC2705234

[6] Li R, Li Y, Fang X, Yang H, Wang J, Kristiansen K. SNP detection for massively parallel whole-genome resequencing. Genome Res. 2009;19: 1124–1132. 10.1101/gr.088013.108 19420381 PMC2694485

[7] McKenna A, Hanna M, Banks E, Sivachenko A, Cibulskis K, Kernytsky A, et al The Genome Analysis Toolkit: a MapReduce framework for analyzing next-generation DNA sequencing data. Genome Res. 2010;20: 1297–1303. 10.1101/gr.107524.110 20644199 PMC2928508

[8] Li H. The Sequence Alignment/Map format and SAMtools. Bioinformatics. 2009;25: 2078–2079. 10.1093/bioinformatics/btp352 19505943 PMC2723002

[9] Danecek P, Auton A, Abecasis G, Albers CA, Banks E, DePristo MA, et al The variant call format and VCFtools. Bioinformatics. 2011;27: 2156–2158. 10.1093/bioinformatics/btr330 21653522 PMC3137218

[10] McLaren W, Pritchard B, Rios D, Chen Y, Flicek P, Cunningham F. Deriving the consequences of genomic variants with the Ensembl API and SNP Effect Predictor. Bioinformatics. 2010;26: 2069–2070. 10.1093/bioinformatics/btq330 20562413 PMC2916720

[11] Wang K, Li M, Hakonarson H. ANNOVAR: functional annotation of genetic variants from high-throughput sequencing data. Nucleic Acids Res. 2010;38: e164 10.1093/nar/gkq603 20601685 PMC2938201

[12] Shihab HA, Gough J, Cooper DN, Stenson PD, Barker GL, Edwards KJ, et al Predicting the functional, molecular, and phenotypic consequences of amino acid substitutions using hidden Markov models. Hum Mutat. 2013;34: 57–65. 10.1002/humu.22225 23033316 PMC3558800

[13] Ng P, Henikoff S. SIFT: Predicting amino acid changes that affect protein function. Nucleic Acids Res. 2003;31: 3812–3814. 12824425 10.1093/nar/gkg509 PMC168916

[14] Adzhubei IA, Schmidt S, Peshkin L, Ramensky VE, Gerasimova A, Bork P, et al A method and server for predicting damaging missense mutations. Nat Meth. 2010;7: 248–249. 10.1038/nmeth0410-248 PMC2855889 20354512

[15] Gonzalez-Perez A, Lopez-Bigas N. Improving the assessment of the outcome of nonsynonymous SNVs with a consensus deleteriousness score, Condel. Am J Hum Genet. 2011;88: 440–449. 10.1016/j.ajhg.2011.03.004 21457909 PMC3071923

[16] Consortium TGP. A map of human genome variation from population-scale sequencing. Nature. 2010;467: 1061–1073. 10.1038/nature09534 20981092 PMC3042601

[17] ESP NG (2013) Exome Variant Server, NHLBI GO Exome Sequencing Project (ESP). Web.

[18] Gaunt TR, Cooper JA, Miller GJ, Day IN, O'Dell SD. Positive associations between single nucleotide polymorphisms in the IGF2 gene region and body mass index in adult males. Hum Mol Genet. 2001;10: 1491–1501. 11448941 10.1093/hmg/10.14.1491

[19] Day IN, Humphries SE. Electrophoresis for genotyping: microtiter array diagonal gel electrophoresis on horizontal polyacrylamide gels, hydrolink, or agarose. Anal Biochem. 1994;222: 389–395. 7864363 10.1006/abio.1994.1507

[20] Thorvaldsdottir H, Robinson JT, Mesirov JP. Integrative Genomics Viewer (IGV): high-performance genomics data visualization and exploration. Brief Bioinform. 2013;14: 178–192. 10.1093/bib/bbs017 22517427 PMC3603213

[21] Davydov EV, Goode DL, Sirota M, Cooper GM, Sidow A, Batzoglou S. Identifying a High Fraction of the Human Genome to be under Selective Constraint Using GERP++. PLoS Comput Biol. 2010;6: e1001025 10.1371/journal.pcbi.1001025 21152010 PMC2996323

[22] Zhang Y, Lundgren T, Renvert S, Tatakis DN, Firatli E, Uygur C, et al Evidence of a founder effect for four cathepsin C gene mutations in Papillon-Lefevre syndrome patients. J Med Genet. 2001;38: 96–101. 11158173 10.1136/jmg.38.2.96 PMC1734811

[23] Stenson PD, Ball EV, Mort M, Phillips AD, Shaw K, Cooper DN. The Human Gene Mutation Database (HGMD) and its exploitation in the fields of personalized genomics and molecular evolution. Curr Protoc Bioinformatics. 2012;Chapter 1: Unit1 13. 10.1002/0471250953.bi0113s39 22948725

[24] Rabbani B, Tekin M, Mahdieh N. The promise of whole-exome sequencing in medical genetics. J Hum Genet. 2014;59: 5–15. 10.1038/jhg.2013.114 24196381

[25] Yu JH, Harrell TM, Jamal SM, Tabor HK, Bamshad MJ. Attitudes of genetics professionals toward the return of incidental results from exome and whole-genome sequencing. Am J Hum Genet. 2014;95: 77–84. 10.1016/j.ajhg.2014.06.004 24975944 PMC4085580

