# Supplementary material for: Proxy Molecular Diagnosis from Whole-Exome Sequencing Reveals Papillon-Lefevre Syndrome Caused by a Missense Mutation in CTSC
Source: PLoS One. 2015 Mar 23;10(3):e0121351. doi: 10.1371/journal.pone.0121351 (PMC4370501; doi:10.1371/journal.pone.0121351)
Supplement: S1 Fig — (DOCX) [file pone.0121351.s001.docx]

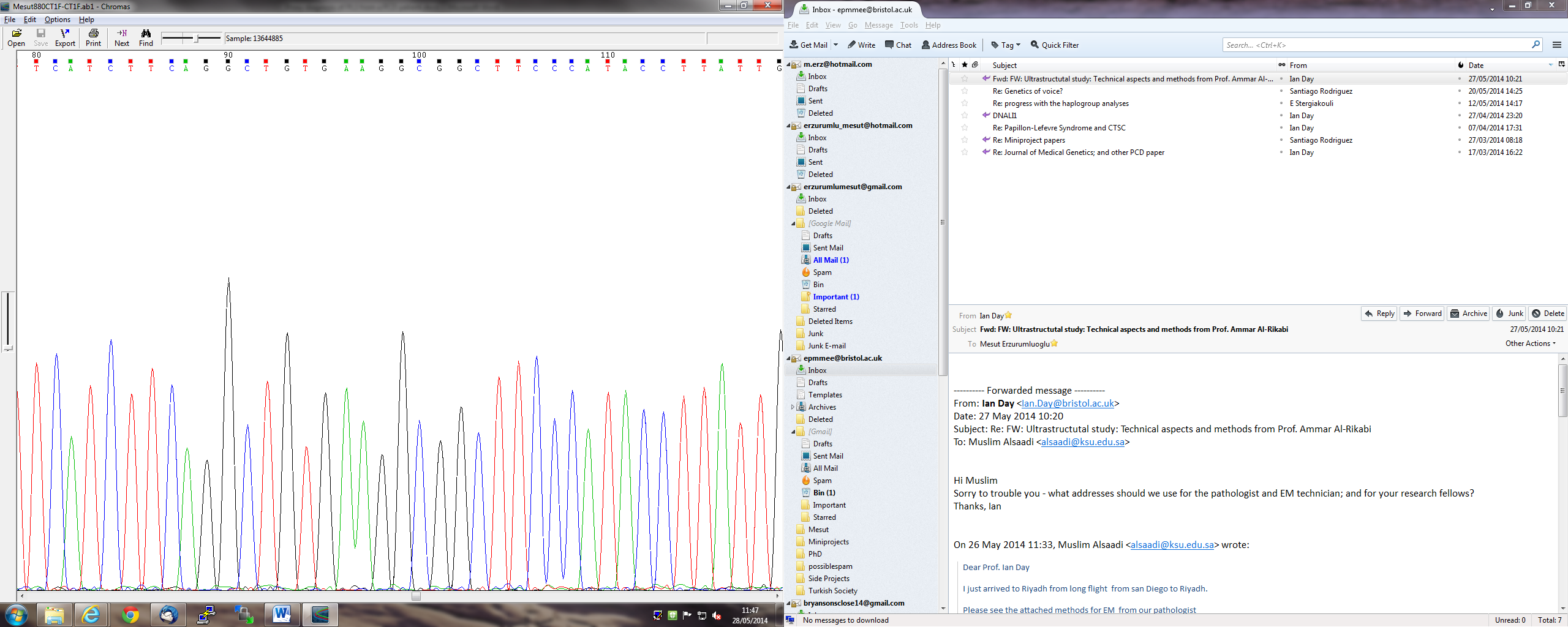

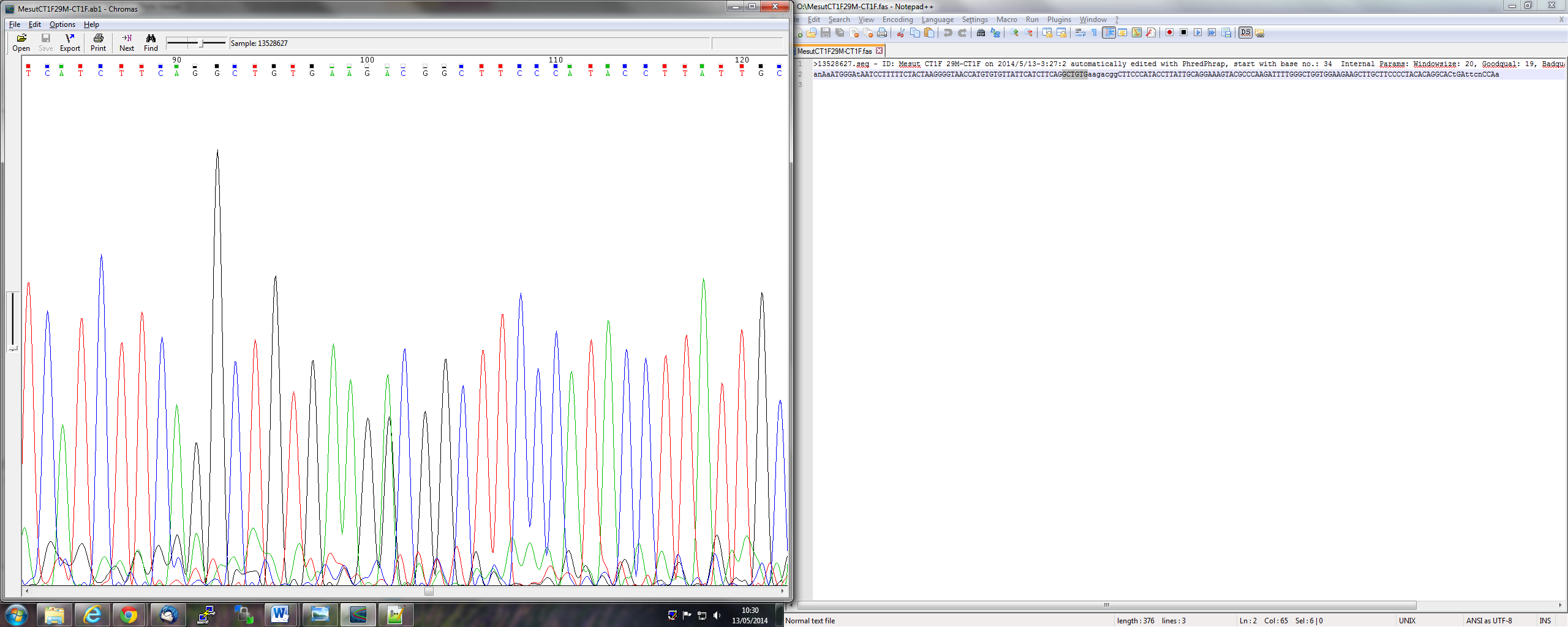


G/A het

**e**


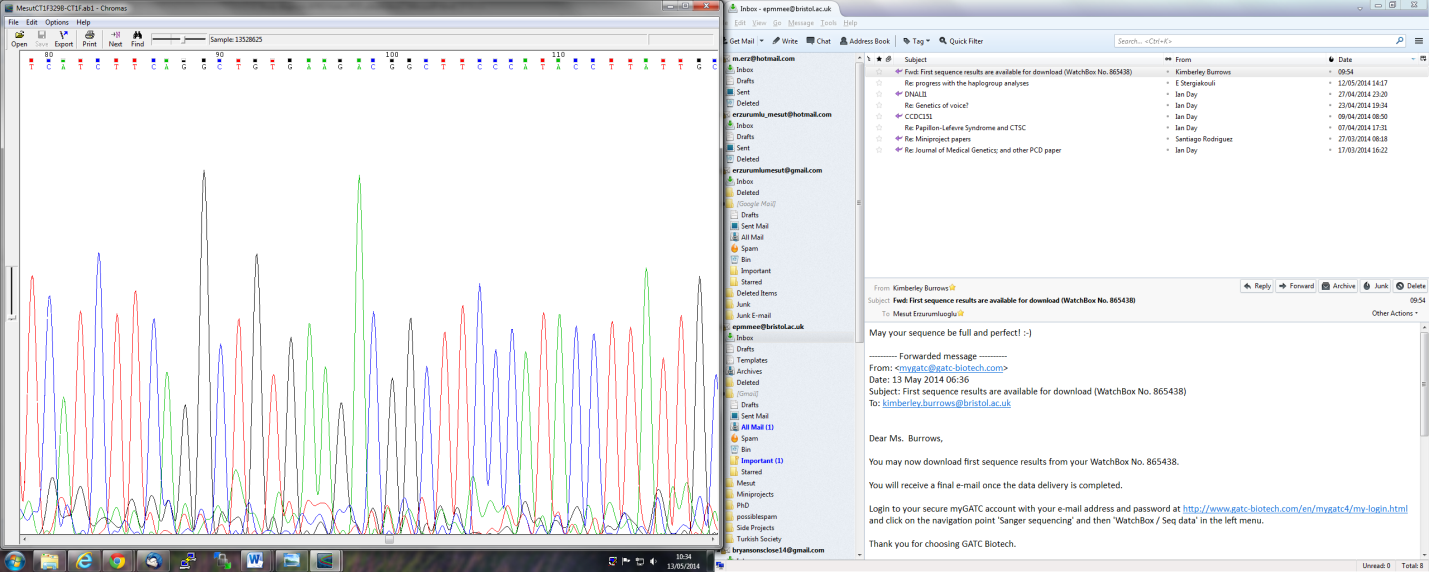


A/A

**b**


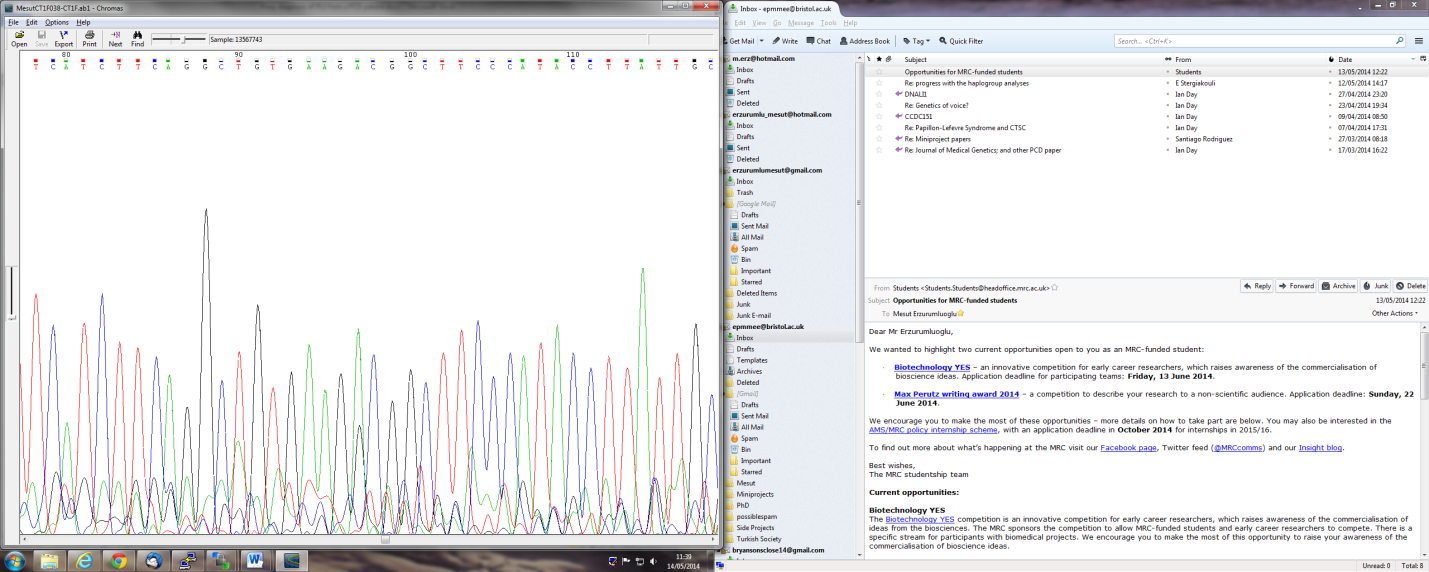


G/A het

**d**


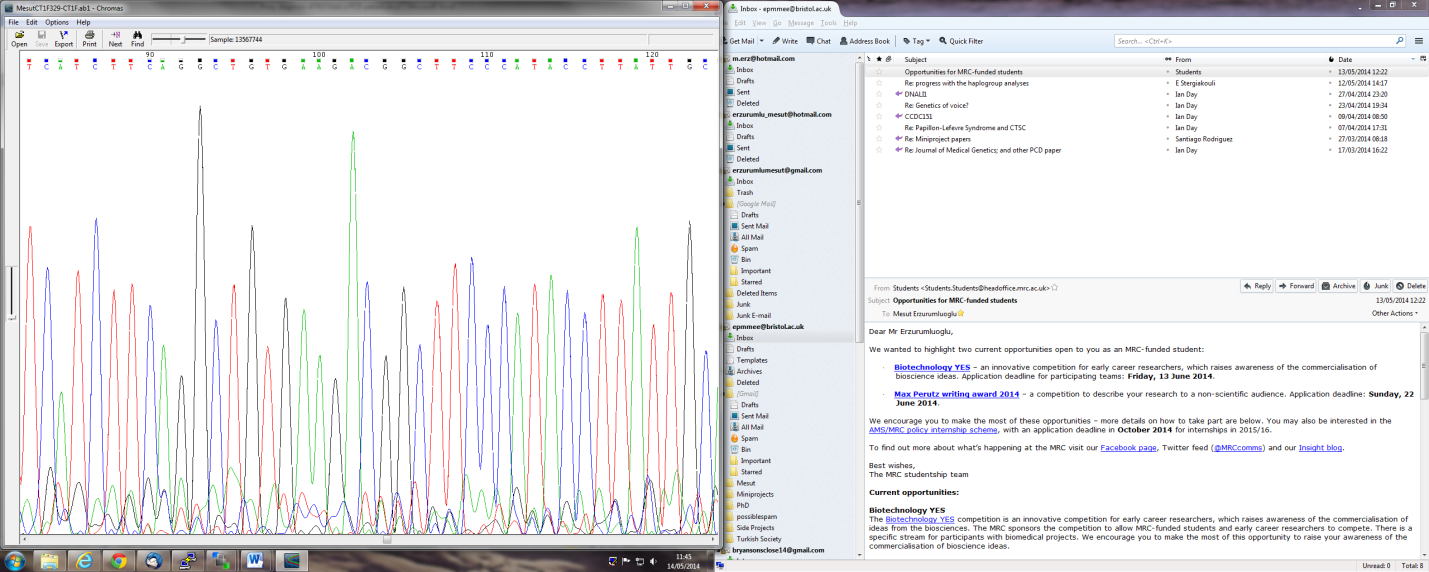


**a**

A/A


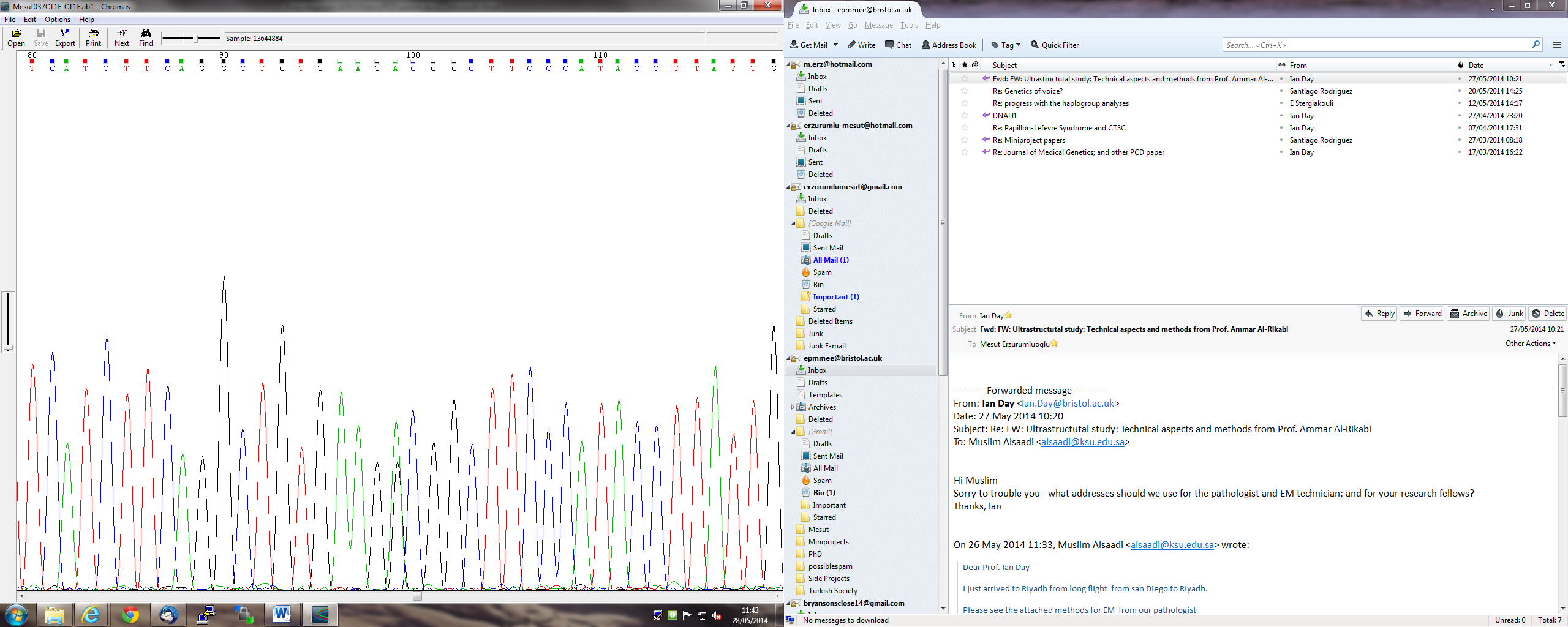


G/A het

**f**

G/G

**c**

**S1 Figure** Confirmation of variant status in other family members using Sanger sequencing: (a) Male proband (b) Affected brother (c) Unaffected sister (d) Father (e) Mother (f) PCD affected (and PLS unaffected) sibling whose WES data was available.
