# Supplementary material for: Proxy Molecular Diagnosis from Whole-Exome Sequencing Reveals Papillon-Lefevre Syndrome Caused by a Missense Mutation in CTSC
Source: PLoS One. 2015 Mar 23;10(3):e0121351. doi: 10.1371/journal.pone.0121351 (PMC4370501; doi:10.1371/journal.pone.0121351)

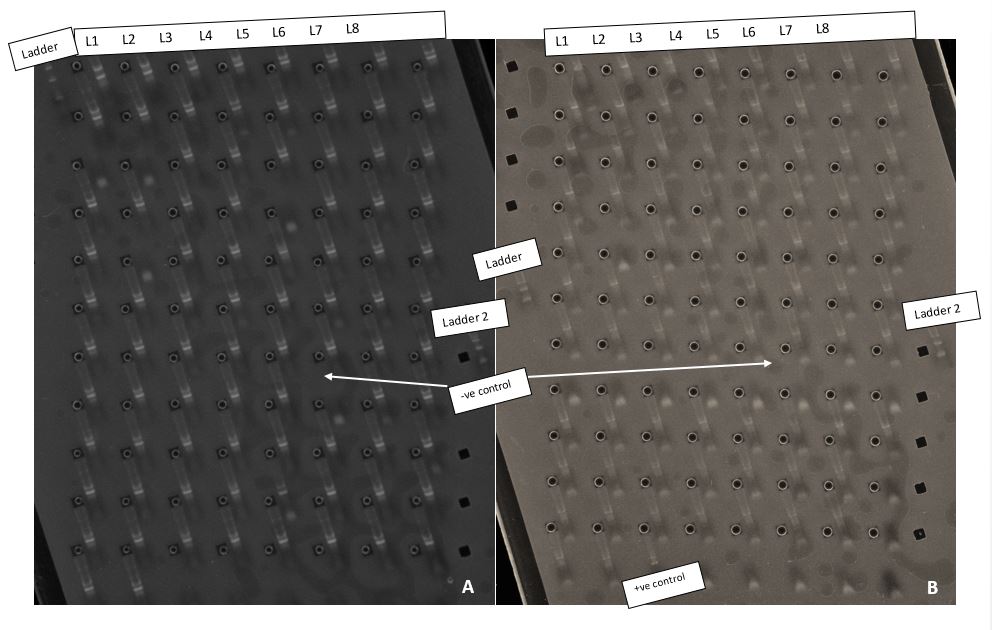


**S2A-F Figure** Screening the local population for the c.899G>A:p.(G300D) variant. 96-well MADGE images reveal that none of the 256 individuals have the causal allele. ARMS-PCR (A,C,E) using wild type primers (B,D,F) using AS primers. A, C and E are complementary to B, D, and F respectively. Ladder’s three bands are 100bp (bottom), 200bp and 300bp (top).


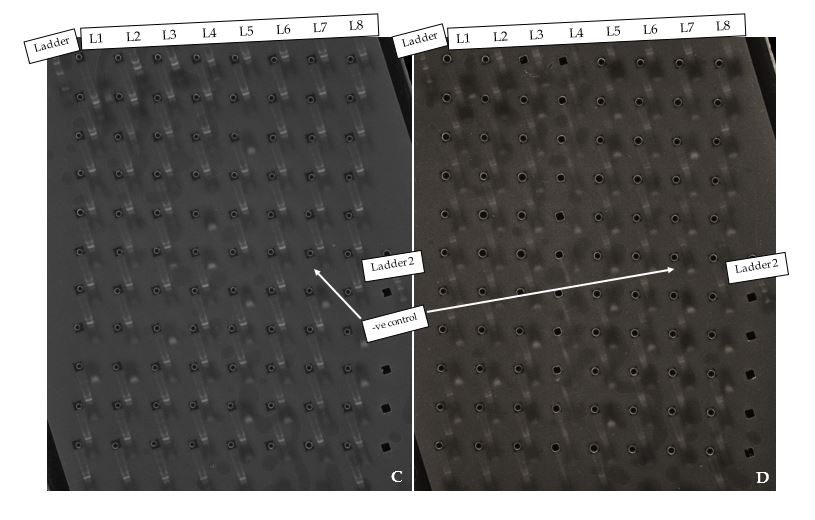


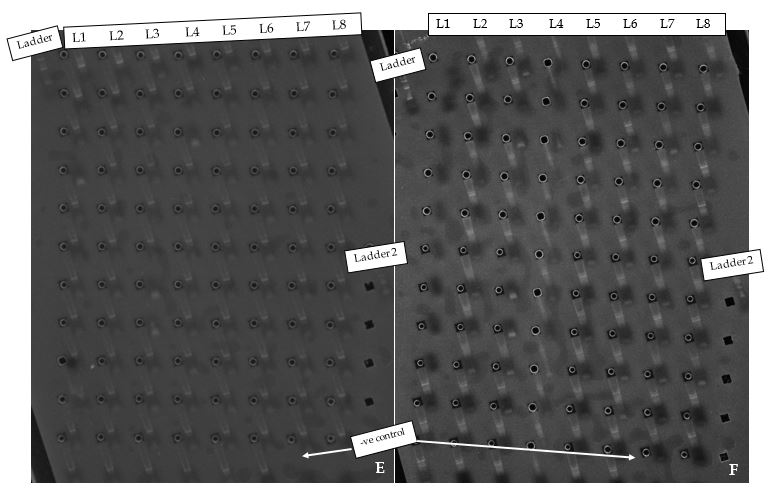

Supplement: S2 Fig — (DOCX) [file pone.0121351.s002.docx]
