# Supplementary material for: Proxy Molecular Diagnosis from Whole-Exome Sequencing Reveals Papillon-Lefevre Syndrome Caused by a Missense Mutation in CTSC
Source: PLoS One. 2015 Mar 23;10(3):e0121351. doi: 10.1371/journal.pone.0121351 (PMC4370501; doi:10.1371/journal.pone.0121351)
Supplement: S1 Table — (DOCX) [file pone.0121351.s003.docx]

| Start | Amino acid sequence | **End** | Entry | Entry Name | **Organism** |
| --- | --- | --- | --- | --- | --- |
| 296 | QGCEGGFPYLIAGKYAQDFGLVEE | 319 | P53634 | CATC_HUMAN | *Homo sapiens* (Human) |
| 295 | QGCDGGFPYLIAGKYAQDFGVVEE | 318 | [P80067](http://www.uniprot.org/uniprot/P80067) | CATC_RAT | *Rattus norvegicus* (Rat) |
| 269 | QGCEGGFPYLIAGKYAQDFGLVEE | 292 | [O97578](http://www.uniprot.org/uniprot/O97578) | CATC_CANFA | *Canis familiaris* (Dog) |
| 296 | QGCEGGFPYLIAGKYAQDFGLVEE | 319 | [F1N455](http://www.uniprot.org/uniprot/F1N455) | F1N455_BOVIN | *Bos taurus* (Bovine) |
| 296 | QGCDGGFPYLIAGKYAQDFGLVEE | 319 | [M3W9M0](http://www.uniprot.org/uniprot/M3W9M0) | M3W9M0_FELCA | *Felis catus* (Cat) |
| 295 | QGCDGGFPYLIAGKYAQDFGVVEE | 318 | [P97821](http://www.uniprot.org/uniprot/P97821) | CATC_MOUSE | *Mus musculus* (Mouse) |
| 289 | QGCDGGFPYLI-GKYIQDFGIVEE | 312 | [Q6P2V1](http://www.uniprot.org/uniprot/Q6P2V1) | Q6P2V1_DANRE | *Danio rerio* (Zebrafish) |
| 291 | QGCEGGFPYLIAGKYVSDYGIVEE | 314 | [F7E2G8](http://www.uniprot.org/uniprot/F7E2G8) | F7E2G8_XENTR | *Xenopus tropicalis* (Western clawed frog) |
| 296 | QGCEGGFPYLIAGKYAQDFGLVEE | 319 | [H2Q4I9](http://www.uniprot.org/uniprot/H2Q4I9) | H2Q4I9_PANTR | *Pan troglodytes* (Chimpanzee) |
| 296 | QGCEGGFPYLIGGKYAQDFGLVEE | 319 | [G3VM46](http://www.uniprot.org/uniprot/G3VM46) | G3VM46_SARHA | *Sarcophilus harrisii* (Tasmanian devil) |
| 242 | QGCDGGFPYLIAGKYTQDFGVVEE | 265 | [F7F2M7](http://www.uniprot.org/uniprot/F7F2M7) | F7F2M7_ORNAN | *Ornithorhynchus anatinus* (Duckbill platypus) |
| 296 | QGCNGGFPYLIAGKYAQDFGLVEE | 319 | [G1T7L0](http://www.uniprot.org/uniprot/G1T7L0) | G1T7L0_RABIT | *Oryctolagus cuniculus* (Rabbit) |
| 296 | QGCEGGFPYLIAGKYAQDFGLVEE | 319 | [G3SMJ4](http://www.uniprot.org/uniprot/G3SMJ4) | G3SMJ4_LOXAF | *Loxodonta africana* (African elephant) |
| 296 | QGCAGGFPYLIAGKYAQDFGLVEE | 319 | [F1STR1](http://www.uniprot.org/uniprot/F1STR1) | F1STR1_PIG | *Sus scrofa* (Pig) |
| 300 | QGCEGGFPYLVAGKYAQDFGVIEE | 323 | [T2MEP6](http://www.uniprot.org/uniprot/T2MEP6) | T2MEP6_HYDVU | *Hydra vulgaris* (Hydra attenuata) |
| --- | QGC*GGFPYL**GKY**D*G**EE |  |  |  |  |

**S1 Table** Local sequence alignment containing the mutated residue from multiple alignment of the *CTSC* gene in different species. The highlighted Glycine (G) residue is found to be highly conserved across many species which have a homologue of the human *CTSC* gene. The alignment was carried out using the Uniprot website’s Blast and Align functions (<http://www.uniprot.org>). The final row shows there is diversity in adjacent and nearby residues (represented by *), however the G residue is highly conserved.
