# Supplementary material for: Proxy Molecular Diagnosis from Whole-Exome Sequencing Reveals Papillon-Lefevre Syndrome Caused by a Missense Mutation in CTSC
Source: PLoS One. 2015 Mar 23;10(3):e0121351. doi: 10.1371/journal.pone.0121351 (PMC4370501; doi:10.1371/journal.pone.0121351)
Supplement: S2 Table — (DOCX) [file pone.0121351.s004.docx]

| **Genomic DNA (5’ to 3’)** | **Allele-specific primer – Mutant (5’ to 3’)** | **Allele-specific primer - Wild type (5’ to 3’)** | **Control primer (5’ to 3’)** | **Common primer (5’ to 3’)** | **Size of PCR amplicons** |
| --- | --- | --- | --- | --- | --- |
| **Wild type** TTCATCTTCAGGCTGTGAAGG  **Mutant**  TTCATCTTCAGGCTGTGAAGA | TTCATCTTCAGGCTGTGAACA | TTCATCTTCAGGCTGTGAACG | AACATGCAAAGAATAATGGAG | AGCTTCATCAGGGCTTCATTG | Control-common = 291bp  AS-common = 207bp |

**S2** **Table** Primers used in ARMS-PCR for genotyping the NM_001814.4:c.899G>A:p.(G300D) variant. The allele-specific (AS) primers have a mismatch at the -2 position (highlighted in grey). The AS and control primers are used as forward primers in PCR; and common primer is a reverse primer.
